# Association between anthraquinone laxatives and colorectal cancer: protocol for a systematic review and meta-analysis

**DOI:** 10.1186/s13643-020-1280-5

**Published:** 2020-01-24

**Authors:** Niccolò Lombardi, Alessandra Bettiol, Giada Crescioli, Valentina Maggini, Eugenia Gallo, Francesco Sivelli, Francesco Sofi, Gian Franco Gensini, Alfredo Vannacci, Fabio Firenzuoli

**Affiliations:** 1 Department of Neurosciences, Psychology, Drug Research and Child Health, Section of Pharmacology and Toxicology, Tuscan Regional Centre of Pharmacovigilance and Phytovigilance, University of Florence, Viale G. Pieraccini, 6, 50139 Florence, Italy; 2 Department of Experimental and Clinical Medicine, University of Florence, Florence, Italy; 3 Research and Innovation Center in Phytotherapy and Integrated Medicine CERFIT, Referring Center for Phytotherapy, Tuscany Region, Careggi University Hospital, Florence, Italy; 4 Permanent Commission for Guidelines, Tuscany Region, Florence, Italy

## Abstract

**Introduction:**

Products containing anthraquinones (AQ) are mainly used as laxatives and have several biological effects. Long-term use of AQ laxatives is associated with an increased risk of serious adverse events (AEs), such as colorectal cancer (CRC). We will systematically synthesize the evidence on the potential association between the use of AQ laxatives and the risk of CRC.

**Methods and analysis:**

We will search MEDLINE, Embase, Scopus, the Cochrane Library, Google Scholar, and Clinicaltrials.gov. To avoid missing any relevant studies, we will search the bibliographies of retrieved papers and recent reviews in the field. Interventions will include products containing oral AQ laxatives, in particular, those derived from rhubarb, senna, cascara, buckhorn, and aloe. Two review authors will independently screen title, abstract, and full texts and will independently extract data from included studies. The primary outcome is the number of participants diagnosed with CRC, while the secondary outcome will be cases of melanosis coli. We will also consider all other AEs reported in the included studies, in particular, intestinal bleeding, alterations of gastrointestinal motility, and potential for dependence. When possible and appropriate, for each outcome, a meta-analysis will be performed.

**Discussion:**

This protocol is prepared in accordance with the Preferred Reporting Items for Systematic Reviews and Meta-Analyses Protocols guidelines. The protocol gives an insight into the scope and parameters for the systematic review to be carried out.

**Systematic review registration:**

PROSPERO CRD42019125414

## Introduction

Anthraquinones (AQs) are found in rhubarb root, Senna leaf and pod, Cascara, Buckhorn, and Aloe, and they are widely used in laxative preparations. AQ laxatives include physcion, chrysophanol, aloe-emodin, rhein, and sennosides. After oral ingestion, AQs are generally metabolized to active aglycones, which exert their laxative effect by damaging epithelial cells, leading directly and indirectly to changes in intestinal absorption, secretion, and motility [1]. In particular, two different mechanisms of action have been proposed: (1) an effect on large intestine motility resulting in accelerated colonic transit, thus reducing fluid absorption, and (2) an effect on secretion processes resulting in enhanced fluid absorption. At a cellular level, one main target is the inhibition of the Cl^−^-channels across colon cells, contributing to the laxative effect [2]. Moreover, Na^+^/K^+^-ATPase pump is inhibited by those 1,8-dihydroxyanthrones/anthraquinones bearing an additional phenolic hydroxyl group [3].

Damaged epithelial cells can be found in the pigmented colonic mucosa, a characteristic of melanosis coli, a condition potentially related to an inappropriate use of AQ laxatives (i.e., more than 2 weeks of treatment). The question whether the melanosis coli predisposes to colorectal cancer (CRC) is controversial [4], and the relationship between the use of AQ laxatives, melanosis coli, and CRC is still debated.

Pre-clinical studies have shown a potential role of AQ laxatives in both the initiation and promotion of tumorigenesis, and studies performed in humans have also suggested tumor promoting activities for these laxatives [5].

CRC is one of the main concerns of modern medicine, representing one of the most common types of cancer worldwide [6]. In both sexes, CRC represents the fourth type of cancer for incidence (6.1% of the total cases) and the first one for mortality (8.2% of the total cancer deaths).

In high-income countries, AQ laxatives are used by 20% of the population [7]. The World Health Organization (WHO) has published monographs on safety, efficacy, and quality control of Aloe, Cassia, Frangula, and Cascara for their use as medicinal plants [8]. In the monographs, it is recommended that products containing AQ glycosides should not be used for longer than 1–2 weeks, due to the possible incidence of serious AEs, such as electrolyte imbalance. Also the European Medicines Agency (EMA) recommends not using AQ laxatives (i.e., those containing rhubarb) for more than 2 weeks. Otherwise, treatment with product containing AQ laxatives should always require medical supervision [9]. In Germany, the Federal Institute for Medicines and Medical Devices has recommended not to use AQ laxatives for prolonged periods [10]. Although research to date has shown that long-term use of AQs does not necessarily lead to serious adverse events (AEs), it may be prudent to use such products only for short-term relief of constipation (e.g., potential for dependence, drug-drug interactions), in particular when they are used as a self-medication treatment [11].

Recently, the European Food Safety Authority (EFSA) provided a scientific opinion on the safety of AQ derivatives [12]. EFSA’s document was triggered by concerns about the possible harmful effects associated with long-term consumption of AQ-containing preparations (i.e., food supplements used as laxatives). In particular, EFSA reviewed existing scientific evidence on the possible link between AQ products intake and adverse health effects (i.e., colorectal cancer) [12]. In their document, the panel of experts concluded that AQs should be considered as genotoxic and carcinogenic unless there are specific data to the contrary. Furthermore, they were unable to provide safety advice on the daily intake of AQ products.

Since there is no clear evidence of the potential association between the use of oral AQ laxatives and the risk of CRC, we aimed to quantify this risk by performing a systematic review and meta-analysis.

## Methods and analysis

This protocol has been written according to the Preferred Reporting Items for Systematic review and Meta-Analysis Protocols guidance [13, 14] and has been registered on PROSPERO (registration ID CRD42019125414).

### Eligibility criteria

Studies will be selected according to the eligibility criteria outlined below.

#### Study designs

We will consider for inclusion both clinical trials and observational cohort studies, either prospective or retrospective. We will also include case-control studies. Observational cross-sectional studies will be excluded. Similarly, we will exclude reviews and meta-analyses, letters to the editor, case reports, case series, and expert opinions.

#### Participants

We will consider studies performed on subjects taking AQs as oral laxatives, excluding studies which include patients with history of any cancer. No restriction on subjects’ age will be applied.

#### Interventions

We will consider the following plant-containing AQ laxatives:

- Senna, syn. Cassia (*Cassia acutifolia*, *C*. *angustifolia*)
- Frangula (*Rhamnus frangula*)
- Cascara (*Rhamnus purshiana*, *Syn*. *Cascara sagrada*)
- Rhubarb (*Rheum officinale*, *R*. *palmatum*)
- Aloe spp. (*Aloe vera*, *syn*. *A*. *barbadensis*, *A*. *ferox*, *A*. *arborescens*)

We will also consider all active AQ compounds, such as: physcion, chrysophanol, rhein, dantron, emodin, aloe-emodin, and senna glycosides (sennoside A and B) [15]. Additional active compounds or interventions containing AQ laxatives, not listed above and detected by screening of retrieved references or in the bibliographies of evaluated studies, will be also considered.

Studies on patients co-treated with more than one abovementioned AQ laxatives will be included as well.

#### Comparators

We will consider studies evaluating the effect of the above mentioned AQ laxatives compared to no treatment and/or compared to non-AQ laxatives.

#### Outcomes

We will include studies evaluating the primary safety outcome “CRC” and/or studies evaluating the secondary safety outcome “melanosis coli”.

In studies evaluating at least one of the abovementioned safety outcomes, we will also consider the following AEs:

1. Gastrointestinal bleeding
2. Alterations in gastrointestinal motility
3. Potential for dependence

We will also consider any other AEs experienced by treated subjects in included studies; AEs will be defined based on authors’ definitions.

#### Timing

There will be no timing restriction. We will define a consumption of AQ laxatives less than 2 weeks as “short-term” use, while “long-term” use will be referred to as consumption longer than 2 weeks.

Regardless of the time of onset, we will include any diagnosis of CRC in patients exposed to AQ laxatives for a period exceeding 2 weeks (“long-term” use). Then we will perform a stratification based on the latency time, taking into account the clinical characteristics of each patient and evaluating the events of CRC on a case-by-case basis. For this purpose, if necessary, we will request data at the single patient level from authors of the included original studies.

#### Setting

There will be no restriction by type of setting.

#### Language

We will include articles written in any language.

### Information sources and search strategy

Electronic searches will be performed in the databases MEDLINE, Embase, Scopus, the Cochrane Library, Google Scholar, and Clinicaltrials.gov.

The MEDLINE search strategy is reported below:

1. (anthraquinon*[tiab] OR anthrachinon*[tiab] OR anthraquinonoid*[tiab] OR carmine*[tiab] OR cascara*[tiab] OR emodin*[tiab] OR senna*[tiab] OR cassia*[tiab] OR frangula*[tiab] OR rhamnus*[tiab] OR rheum[tiab] OR rumex*[tiab] OR rhubarb*[tiab] OR aloe*[tiab] OR sennosid*[tiab] OR physcion*[*tiab*] OR chrysophanol*[*tiab*] OR rhein*[*tiab*] OR dantron*[tiab] OR laxativ*[tiab] OR propulsiv*[tiab] OR "anthraquinones"[Mesh] OR "laxatives"[Mesh])

2. (cancer*[tiab] OR carcinom*[tiab] OR neoplas*[tiab] OR tumor[tiab] OR tumoral[tiab] OR tumorigen*[tiab] OR malignan*[tiab] OR oncogen*[tiab] OR mutagen*[tiab] OR oncolog*[tiab] OR "neoplasms"[Mesh]) AND (intestinal*[tiab] OR colon*[tiab] OR rectal*[tiab] OR colorectal*[tiab])
3. ("Hyperpigmentation"[Mesh] OR melanosis*[tiab] OR pigment*[tiab])
4. *(case reports[ptyp] OR comment[sb] OR editorial[ptyp] OR guideline[ptyp] OR meta-analysis[ptyp] OR practice guideline[ptyp] OR review[ptyp] OR systematic[sb])*
5. *(french[lang] OR spanish[lang] OR german[lang] OR chinese[lang] OR hindi[lang] OR arabic[lang] OR italian[lang] OR turkish[lang] OR swedish[lang] OR danish[lang])*
6. *2 OR 3*
7. *1 AND 6*
8. *7 NOT 4 NOT 5*

The MEDLINE search strategy will be adapted to the syntax and subject headings of the Embase, Scopus, the Cochrane Library, and Google Scholar.

Records will be retrieved on the same day from all sources.

The search strategy will be updated toward the end of the review, after being validated to ensure that the MEDLINE strategy retrieves a high proportion of eligible studies found through any means and indexed in MEDLINE.

### Study records

#### Data management

Retrieved records will be managed using the software EndNote™.

#### Selection process

Two review authors will independently screen the extracted records. The two review authors will independently identify studies for inclusion by screening titles and abstracts yielded by search, eliminating those deemed irrelevant. We will retrieve full-text articles for all references that at least one of the review authors will identify for potential inclusion.

We will select studies for inclusion on the basis of review of full-text articles. We will resolve discrepancies through discussion.

Neither of the review authors will be blind to the journal titles or to the study authors or institutions.

#### Data collection

Two review authors will independently extract data from the included studies.

Data abstracted will include demographic information, methodology, intervention details, all reported clinically relevant conditions, and outcomes. Data will be extracted at the trial arm level. We will resolve discrepancies between authors through discussion.

### Data items

Extracted data will include the name of the study authors and year of publication, the study design and characteristics (including single or double blinding and randomization), the country in which participants were recruited, and eventual funding sources.

As for the population, we will extract the subjects’ age, and clinically relevant comorbidities.

As for the intervention and the comparator, we will extract the active principle of the experimental intervention, its route of administration, the treatment dosage, and the duration of treatment.

We will extract the number of randomized participants, the number of participants included in the analysis, the number of participants with events for binary outcomes, effect size measurements (i.e., odds ratio (OR)) and variables entering the multivariable model as potential confounders, if appropriate. Whenever possible, we will use results from an intention-to-treat analysis.

### Outcomes and prioritization

The primary safety outcome will be the number of subjects diagnosed with “CRC”, out of the total number of treated patients.

The secondary safety outcome will be the number of cases of “melanosis coli”, out of the total number of treated patients.

For all outcomes, where OR and related confidence intervals (CIs) are reported, these will be transformed to absolute numbers.

Any AE, if present, will be identified based on specific authors’ definitions and will be classified using the MedDRA classification, according to preferred terms (PT) and system organ class (SOC) classification [16].

### Risk of bias

Two review authors will independently assess the included studies for bias. To assess the risk of bias of included randomized controlled trials, we will follow the Cochrane Handbook for Systematic Reviews of Interventions [17]. Specifically, we will assess the risk of bias for the following domains: selection (random sequence generation; allocation concealment), performance (blinding of participants and personnel), detection (blinding of outcome), attrition (incomplete outcome data), reporting (selective reporting), and other unclear bias.

To assess the risk of bias of observational studies, we will follow the Newcastle-Ottawa Quality Assessment Scale [18]. Specifically, for included cohort studies, we will consider the following domains: selection (representativeness of the exposed cohort, selection of the non-exposed cohort, ascertainment of exposure, absence of outcome of interest at start of study), comparability, and outcome (assessment of outcome, appropriate length of follow-up, adequacy of follow-up of cohorts).

For each domain in the two tools, we will describe the procedures undertaken for each study, including verbatim quotes. A judgment as to the possible risk of bias on each domain will be made from the extracted information, rating from “low-risk” to “high-risk”.

The judgements will be made independently by two review authors; disagreement will be resolved first by discussion and then by consulting a third author.

We will compute graphic representations of potential bias within included studies, using the software RevMan 5.3 (Review Manager 5.3).

### Data synthesis

If studies are sufficiently homogeneous in terms of design and comparator, we will synthesize results using a meta-analysis [14].

#### Measures of treatment effect

All considered outcomes are based on dichotomous data. According to the assessment of statistical heterogeneity, if appropriate, for all considered outcomes, we will perform a meta-analysis using a random-effects model within a frequentist framework. We will calculate pooled ORs combining the estimates reported in each study using random-effects Mantel-Haenszel method.

For all other AEs, no quantitative synthesis will be performed, and the proportions of each reported AE will be described at study level.

#### Unit of analysis issues

All analysis will be conducted per trial arm, rather than at individual patient level.

#### Dealing with missing data

Study authors will be contacted to obtain the missing data. If missing data cannot be obtained, the study will be excluded from the related analysis.

#### Assessment of heterogeneity

We will evaluate the clinical heterogeneity by considering the variability in participants’ features among studies and in study characteristics (study design, intervention, follow-up).

We will evaluate statistical heterogeneity across studies using the I-squared and Cochran’s Q tests, and publication bias using plots of standard error against effect estimate (bias is likely to cause asymmetry in such plots) or using formal tests such as Egger one or similar.

If high levels of heterogeneity exist (*I*-squared ≥ 50% or *P* < 0.1), we will try to explain the source of heterogeneity by conducting subgroup or sensitivity analysis.

### Subgroup and sensitivity analysis

If possible, subgroup analysis will be conducted for different AQ compounds, daily dosages, and duration of treatment (i.e., short- or long-term use).

Additional subgroup analysis will be performed, if appropriate, according to the clinical characteristics of patients in included studies.

We will conduct a sensitivity analysis including only clinical trials versus only observational studies. If possible, a second sensitivity analysis will be performed including only high-quality clinical trials.

### Meta-biases

To determine whether reporting bias is present in included clinical trials, we will evaluate whether the protocol of the clinical trial has been published before recruitment of study patients. Specifically, for studies published after July 2005, we will screen the Clinical Trial Register at ClinicalTrials.gov. We will evaluate whether selective reporting of outcomes is present (outcome reporting bias). The potential for reporting bias will be evaluated using funnel plots (if ≥ 10 studies are present).

### Confidence in cumulative estimate

The quality of evidence will be judged using the Grading of Recommendations Assessment, Development and Evaluation working group (GRADE) scale, considering the domains of risk of bias, consistency, directness, precision, and publication bias. Quality will be adjudicated as high, moderate, low, or very low [17].

## Discussion

To the best of our knowledge, this systematic review and meta-analysis will provide a comprehensive narrative synthesis and quantitative estimate of the risk of CRC in subjects exposed to a long-term treatment with AQ laxatives.

The pooled estimate will guide clinicians and policymakers in informing patients and governments about the risk associated to the use of products containing AQ laxatives. This risk could be greater for self-administered products that are easily available without a medical prescription.

Moreover, it will provide an estimate of the future global CRC burden in the context of the complementary and alternative medicine. Importantly, this systematic review will enable the identification of clinical, epidemiological, and public health gaps, thus outlining directions for further investigation.

The findings from the review will be disseminated in a peer-reviewed journal, and we will recommend or carry out research to bridge the identified gaps.

### Strengths and limitations

A major strength of our study is the comprehensive review within six major databases in order to include all potential articles. Limitations include the heterogeneity in the sample size of the retrieved studies and quality of the study design. Furthermore, prospective studies with a sufficient follow-up period to observe the occurrence of CRC may be lacking.

## Ethics and dissemination

There is no primary data collection involved in this study, thus research ethics approval is not required. Results will be disseminated by release of findings in a peer-reviewed scientific journal, and by abstracts and speeches at international meetings and congresses.
